# Wastewater-to-biofuels and bioproducts through integrated anaerobic digestion, bioelectrochemical systems, and algal biorefineries: a systematic review of techno-economic and life-cycle evidence

**DOI:** 10.1186/s13068-026-02778-y

**Published:** 2026-05-30

**Authors:** Isnanik Juni Fitriyah, Sulistyo Saputro, Sajidan Sajidan

**Affiliations:** 1 Universitas Sebelas Maret, Surakarta, Indonesia; 2 Universitas Negeri Malang, Malang, Indonesia

**Keywords:** Wastewater-to-biofuels, Anaerobic digestion, Bioelectrochemical systems, Algal biorefineries, Techno-economic and life-cycle assessment

## Abstract

Wastewater management is increasingly shifting from conventional pollution control toward resource recovery systems that support clean water access, renewable energy production, and circular bioeconomy development. This systematic review synthesizes the literature on wastewater-to-biofuels and bioproducts through the integration of anaerobic digestion, bioelectrochemical systems, and algal biorefineries, with particular attention to techno-economic analysis and life-cycle assessment evidence. A systematic literature review was conducted on peer-reviewed studies published between 2005 and 2025, resulting in 90 selected articles that were analyzed through thematic and comparative synthesis. The findings show a clear transition from single-technology optimization toward integrated systems based on the water–energy–nutrient nexus. Anaerobic digestion remains the most mature pathway for organic load reduction and biogas production, while bioelectrochemical systems provide additional opportunities for electricity and hydrogen recovery. Algal biorefineries strengthen nutrient recovery and expand the production of biomass-based bioproducts. Comparative synthesis indicates that integrated systems can outperform single technologies when organic load, nutrient availability, process compatibility, operational scale, and product valorization are aligned. Integration may become counterproductive when additional capital cost, operational complexity, energy demand, or downstream processing burdens exceed the value of recovered resources. The TEA–LCA synthesis demonstrates that technology selection should be guided by trade-offs between economic feasibility and environmental performance rather than by technical efficiency alone. This review contributes a decision-oriented synthesis for selecting single or integrated wastewater-to-biofuels configurations that are technically feasible, economically viable, and environmentally sustainable.

## Introduction

Wastewater management is increasingly being repositioned from a linear pollution-control activity toward a resource recovery strategy that supports clean water access, renewable energy production, and circular resource use [51, 56]. Within the SDG 6 agenda, wastewater-to-biofuels systems are relevant because they combine water quality improvement with energy and nutrient recovery [68]. This review uses SDG 6 as the policy frame, while the circular economy and water–energy–nutrient nexus are used as analytical lenses to examine how anaerobic digestion, bioelectrochemical systems, and algal biorefineries can be integrated into sustainable resource recovery systems [9, 27, 36, 42, 62]. Globally more than 2.2 billion people still do not have access to safely managed sanitation services, and about 80% of domestic wastewater in developing countries is discharged directly into the environment without adequate treatment [56]. The wastewater sector is estimated to account for about 3–5% of total global greenhouse gas emissions, with significant contributions from methane (CH₄) and nitrogen oxides (N₂O) having global warming potential of about 28–34 times and 265–298 times higher than carbon dioxide (CO₂) over a 100-year time horizon [62]. In terms of chemical characteristics, domestic wastewater generally contains chemical oxygen demand (COD) of 250–1000 mg/L, total nitrogen of around 20–85 mg/L, and total phosphorus of 4–15 mg/L, which if not managed can trigger eutrophication and degradation of water body quality [68]. Ironically, each 1 kg of COD is theoretically capable of producing about 0.35 m^3^ of methane, with a methane calorific value of about 35.8 MJ/m^3^, so the potential for chemical energy in urban wastewater is estimated to reach 1.5–2 times the operational energy needs of conventional treatment plants if utilized through efficient conversion technology [4]. However, more than 70% of wastewater treatment plants in various countries still focus on reducing pollutant loads alone and have not integrated energy and nutrient recovery systems. The disparity between the potential of energy and available materials with management practices that are still linear underscores the urgency of developing an integrated wastewater-to-biofuels system that is able to simultaneously overcome the water crisis, improve energy security, and reduce carbon emissions within the framework of sustainable development.

A number of previous studies have explored the potential of anaerobic digestion as a key technology in the conversion of wastewater organic matter into biogas. A comprehensive review by [50] shows that anaerobic digestive systems are able to reduce chemical oxygen demand (COD) by more than 80% while producing methane with high energy conversion efficiency, especially in modern reactor configurations such as UASB and anaerobic membrane bioreactors. A study by [74] in the context of bioelectrochemical systems, emphasizes that microbial fuel cells (MFCs) and microbial electrolysis cells (MEC) are not only effective in lowering the organic load of wastewater, but are also capable of producing electricity and hydrogen as value-added by-products, although challenges related to power density, electrode cost, and long-term stability remain barriers to commercialization. [57] stated that the wastewater-based algae biorefinery approach allows the simultaneous use of nitrogen and phosphorus for the production of microalgae biomass which can then be converted into biodiesel, bioethanol, and other high-value bioproducts, thus creating a more circular waste treatment model.

Although the literature on anaerobic digestion, bioelectrochemical systems, and algae biorefineries has grown rapidly, there are some important gaps that have not been comprehensively addressed. First, the integrative gap, which is the lack of studies that review the integration of the three technologies in an integrated value chain based on wastewater resource recovery. Second, the evaluative gap, in the form of a limited study that simultaneously combines techno-economic analysis and life-cycle assessment to assess the trade-offs between financial benefits, emission reduction, and other environmental impacts. Third, the policy and implementation gap, where the relationship between technological innovation and the achievement of SDG 6 targets has not been systematically analyzed in the context of water governance and sustainable energy transition. The novelty of this study lies in its holistic Systematic Literature Review (SLR) approach, which not only compares the performance of each technology, but also evaluates the potential for systemic integration, operational synergies, and long-term sustainability implications based on the perspective of the circular economy and sustainable development.

Based on this background, this research is designed using the Systematic Literature Review (SLR) approach to present a comprehensive and structured scientific synthesis of the wastewater-to-biofuels and bioproducts approach in supporting the achievement of SDG 6 through the integration of anaerobic digestion, bioelectrochemical systems, and algae biorefineries. Methodologically, this study aims to: (1) systematically identify, select, and evaluate reputable scientific publications that discuss the technical performance of each technology in the context of wastewater treatment and bioenergy production; (2) map research trends, integrative approaches, and system configuration models developed in the literature; (3) synthesize findings related to techno-economic analysis and life-cycle assessment aspects to understand economic feasibility and environmental implications comparatively; and (4) identify research gaps, inconsistencies in findings, and opportunities for the development of integrative models based on the circular economy.

## Literature review

### SDG 6, circular economy, and the water–energy–nutrient nexus

SDG 6 provides the policy context for sustainable wastewater management by emphasizing water quality improvement, pollution reduction, wastewater reuse, and safe sanitation. In this review, SDG 6 is not treated as a separate thematic outcome, but as a guiding sustainability frame for assessing how wastewater-to-biofuels systems contribute to cleaner water and resource recovery [51, 56]. The circular economy and water–energy–nutrient nexus provide the conceptual basis for evaluating integrated wastewater-to-biofuels systems. The circular economy highlights the conversion of waste streams into reusable resources, while the nexus perspective emphasizes the interdependence between wastewater treatment, renewable energy production, and nutrient recovery [27, 36]. These concepts support the analytical focus of this review by linking anaerobic digestion, bioelectrochemical systems, and algal biorefineries to broader sustainability outcomes [42, 57, 62, 70].

### Anaerobic digestion and bioelectrochemical systems in bioenergy production

Anaerobic digestion has long been recognized as the main technology in the conversion of wastewater organic matter into biogas, with high efficiency of COD reduction and methane production [32, 34]. Innovations such as anaerobic membrane bioreactors (AnMBR) improve process stability and energy efficiency [48]. Meanwhile, bioelectrochemical systems such as microbial fuel cells (MFCs) and microbial electrolysis cells (MECs) allow the simultaneous production of electricity or hydrogen with waste treatment [52, 69]. According to [43] the technology offers the potential for direct energy conversion from organic substrates, although it still faces challenges related to the cost of electrode materials and commercial scale. The combination of these two approaches expands the opportunities for the production of wastewater-based bioenergy.

### Algae-based biorefineries for the production of biofuels and bioproducts

Algae biorefineries utilize nitrogen and phosphorus content in wastewater for the growth of microalgae biomass which can then be converted into biodiesel, bioethanol, or value-added bioproducts. [52] show that microalgae are able to absorb nutrients efficiently while producing significant amounts of lipids for biodiesel production. [51] also emphasize the role of microalgae in wastewater treatment as well as the mitigation of carbon emissions through photosynthesis. [62] underline that the success of algae biorefinery systems relies heavily on value chain optimization and integration with low-cost nutrient sources such as wastewater. Thus, this approach not only supports the production of biofuels, but also expands the diversification of bioproducts in a bio-based economy.

### Integration of wastewater-to-biofuels technology and techno-economic evaluation and life-cycle assessment

The integration of anaerobic digestion, bioelectrochemical systems, and algae biorefineries in one integrated system is increasingly receiving attention as a strategy to increase resource recovery efficiency. [68] state that the incorporation of biological and electrochemical technologies can improve the clean energy efficiency of wastewater treatment plants. In terms of economic feasibility, [3] suggest that techno-economic analysis (TEA) is important for assessing investment feasibility and operational cost sensitivity. [52] emphasize that a life-cycle assessment (LCA) is needed to thoroughly evaluate environmental impacts, including greenhouse gas emissions and potential eutrophication. Therefore, the integration of technology supported by TEA and LCA evaluations is key in formulating an economically viable and environmentally sustainable wastewater-to-biofuels system.

### Frame of mind

**Fig. 1:**
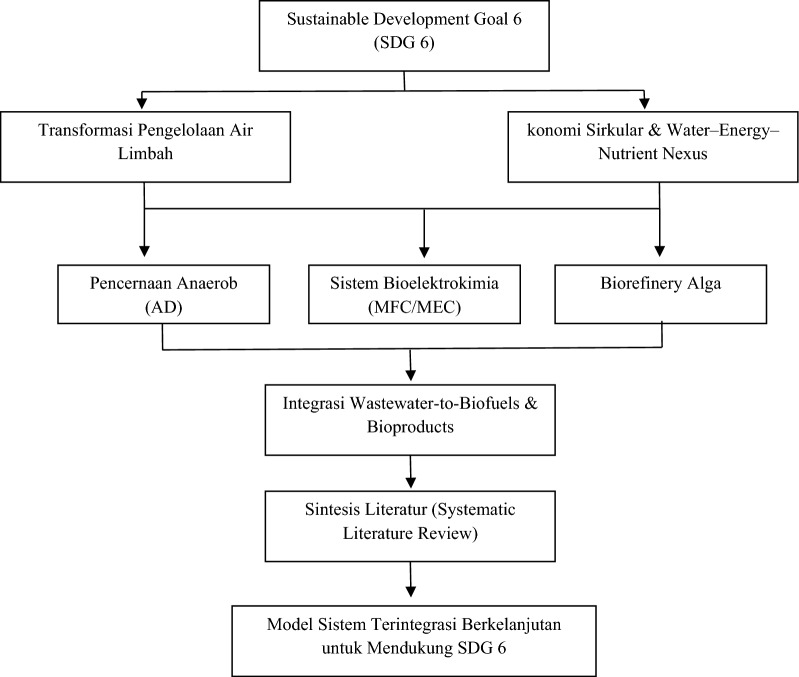
Frame of mind. **Source:** Primary Data (2026)

Based on Fig. 1, the framework of this research shows a conceptual flow starting from Sustainable Development Goal 6 (SDG 6) as a global context that encourages the transformation of wastewater management toward a resource recovery-based approach. This transformation is strengthened by the cornerstone of the circular economy and the concept of the water–energy–nutrient nexus which emphasizes the linkage between water management, energy production, and sustainable use of nutrients. At the operational level, the framework positions three key technologies anaerobic digestion (AD), bioelectrochemical systems (MFC/MEC), and algae biorefinery as strategic components in the conversion of wastewater into biofuels and bioproducts. The three technologies were then integrated into an integrated system of wastewater-to-biofuels and bioproducts which was analyzed through a literature synthesis based on a systematic literature review (SLR). This synthesis stage serves to map empirical findings, identify research gaps, and evaluate potential systemic integration. The final output of this framework is a sustainable integrated systems model that supports the achievement of SDG 6 through improved energy efficiency, nutrient recovery, and comprehensive reduction of environmental impact.

## Research methods

### Research design

This study uses a structured, transparent, and replicative systematic literature review (SLR) approach to identify, evaluate, and synthesize scientific findings related to the integration of anaerobic digestion, bioelectrochemical systems, and algae biorefineries in the framework of wastewater-to-biofuels and bioproducts to support SDG 6 (J. W. [15]). The SLR approach was chosen because it allows systematic screening of the literature based on clear protocols, thereby reducing selection bias and improving the validity of the synthesis of findings. The research process follows the main stages, namely literature identification, initial screening, feasibility evaluation based on inclusion–exclusion criteria, as well as synthesis and comprehensive analysis of selected articles (J. W. [14]).

### Data sources

The data sources for this research come from internationally reputable scientific databases that have a wide scope and high indexed quality, such as Scopus, Web of Science, and ScienceDirect. The databases were chosen because they provide peer-reviewed publications relevant to the fields of wastewater treatment technology, bioenergy, circular economy, as well as sustainability evaluation based on techno-economic analysis (TEA) and life-cycle assessment (LCA) (J. [13]). The articles used are from reputable international journals published within a certain period of time to ensure the relevance and cutting-edge development of the technology being studied.

### Keywords used

The literature search process is carried out using a combination of Boolean operator-based keywords to obtain specific and relevant results. The key keywords used include: "wastewater-to-biofuels", "wastewater resource recovery", "anaerobic digestion", "bioelectrochemical systems", "microbial fuel cells", "microbial electrolysis cells", "algal biorefinery", "circular economy", "techno-economic analysis", "life-cycle assessment", and "SDG 6". The keyword combination was compiled using AND and OR operators to ensure comprehensive literature coverage while remaining focused on technology integration and sustainability evaluation.

### Inclusion and exclusion criteria

**Fig. 2:**
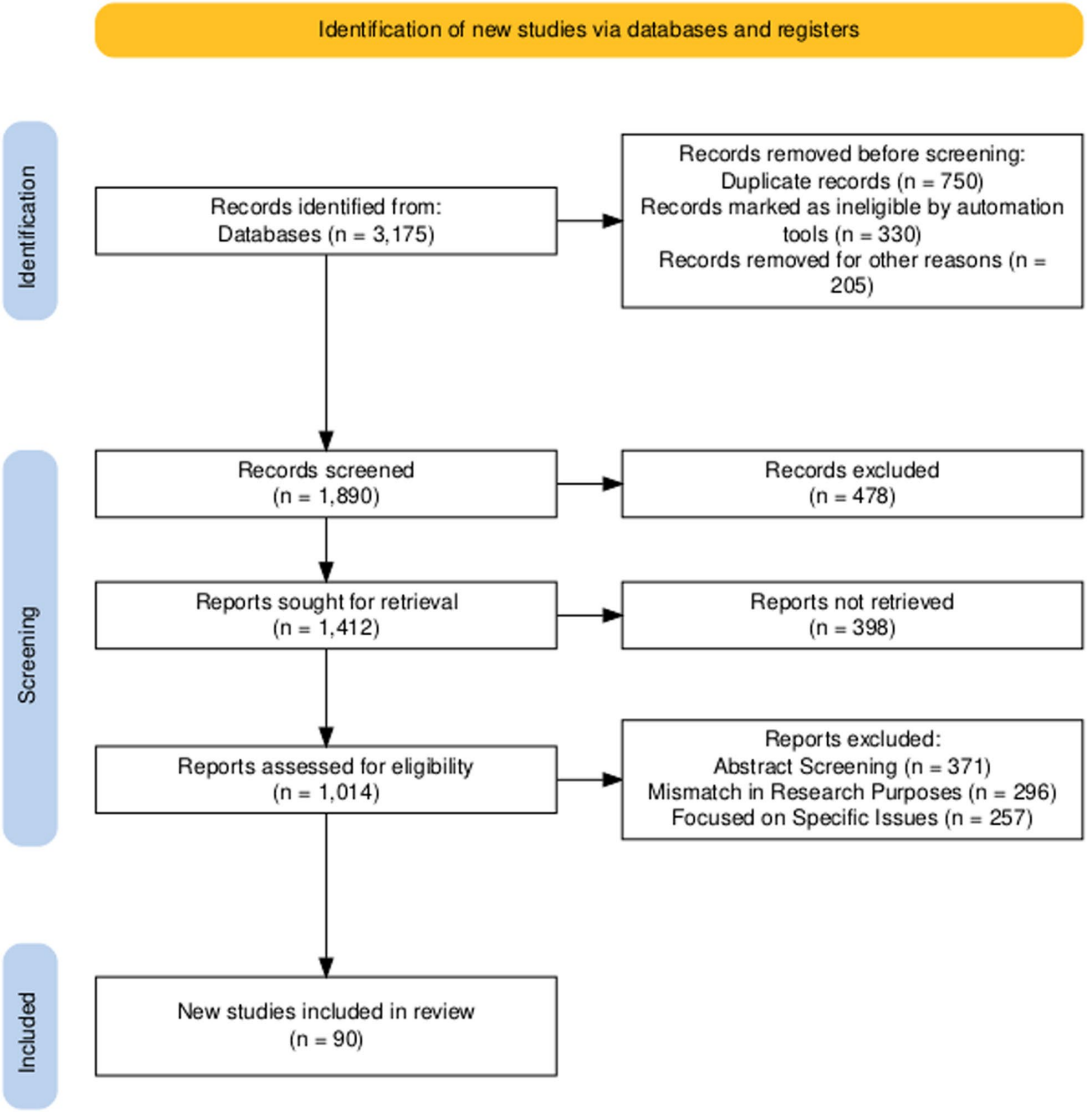
Flowchart of identification, screening, eligibility, and inclusion of studies. **Source:** PRISMA Database (2025)

The determination of articles is carried out based on selection criteria designed to ensure the quality, relevance, and consistency of literature findings. The selected article must be directly related to the topic of integrating wastewater treatment technology into biofuels or bioproducts and must discuss technical, economic, or environmental aspects [31]. The selection process is carried out through the stages of filtering titles, abstracts, and full texts to ensure the suitability of the research substance. The literature selection process in this study was carried out systematically to ensure transparency and clear replication in accordance with the PRISMA guidelines:

Based on Fig. 2, a total of 3175 initial records were identified from various databases before the deduplication and automatic filtering process was carried out. After going through the stages of screening titles, abstracts, and evaluating the suitability of research objectives, the number of studies that met the inclusion criteria was significantly reduced until there were 90 articles that were analyzed in depth. This flow shows that the research has gone through a strict and systematic selection process so that the quality and relevance of the literature used can be accounted for academically. The inclusion and exclusion criteria used are as follows:

Inclusion criteria:

1. Peer-reviewed international journal articles.
2. Published in the period 2005–2025 to represent modern technological developments in the field of bioenergy and wastewater treatment. This period was chosen because since the mid-2000s there has been a significant acceleration in advanced generation anaerobic digestion research, the development of microbial fuel cells and microbial electrolysis cells, as well as the increasing integration of techno-economic analysis (TEA) and life-cycle assessment (LCA) approaches in the evaluation of sustainability systems.
3. Discuss anaerobic digestion, bioelectrochemical systems, or algal biorefineries in the context of wastewater.
4. Examine aspects of system integration, techno-economic analysis (TEA), or life-cycle assessment (LCA).
5. Published in a time span relevant to the development of cutting-edge technology.

Exclusion criteria:

- 1. Non-peer-reviewed articles (proceedings without review, popular reports, opinions).
- 2. Studies that do not focus on wastewater as the main source of raw materials.
- 3. Articles that only discuss theoretical aspects without empirical data or evaluation of systems.
- 4. Duplicate publications in different databases.

## Data analysis techniques

The data analysis technique is carried out through a thematic and comparative synthesis approach to articles that have passed the selection stage. Each article was analyzed based on key variables which included the type of technology, performance parameters (energy efficiency, COD reduction, biofuel production), system integration model, techno-economic analysis results, and environmental impact indicators from life-cycle assessments. Furthermore, research trends are mapped, technology integration patterns are identified, and research gap analysis that appears in the literature. The synthesis process is carried out in a narrative-analytical manner to connect findings between studies and produce an integrative conceptual framework that supports the development of a sustainable wastewater-to-biofuels system model in the context of achieving SDG 6.

## Results and discussion

### Research results

**Fig. 3:**
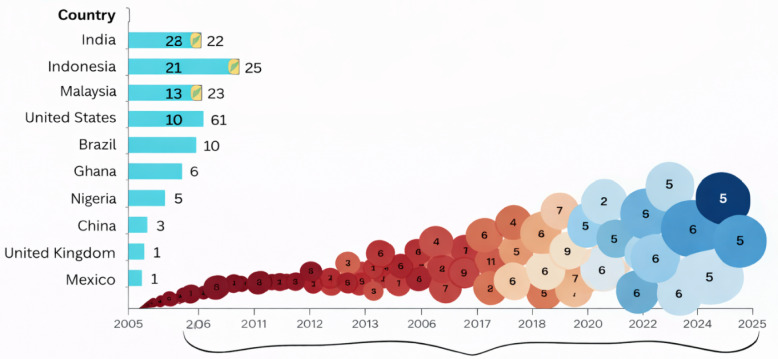
Temporal and geographical distribution of wastewater-to-biofuels research published between 2005 and 2025 ** Source:** Primary Data (2026)

The reviewed literature indicates a clear shift from single-technology optimization toward integrated wastewater-to-biofuels systems. Early studies mainly emphasized the technical efficiency of anaerobic digestion, microbial fuel cells, microbial electrolysis cells, or algal cultivation as separate processes. Recent studies show a stronger interest in hybrid configurations that combine organic matter degradation, direct energy conversion, nutrient recovery, and bioproduct diversification within a circular economy framework. This shift suggests that the central issue in current wastewater-to-biofuels research is not only whether a technology can produce energy, but whether different technologies can be integrated without increasing economic, operational, or environmental burdens. Comparative synthesis shows that integrated systems often outperform single technologies when the wastewater stream contains sufficient organic matter, recoverable nutrients, and stable hydraulic loading. For example, anaerobic digestion provides reliable methane production and organic load reduction, while algal biorefineries can utilize digestate nutrients for biomass production. Bioelectrochemical systems can recover additional energy from residual organic substrates. Under these conditions, integration improves resource recovery efficiency and reduces residual waste streams. By contrast, integration does not automatically improve system performance. Integrated configurations may become less efficient when wastewater has low organic strength, nutrient imbalance, unstable influent characteristics, or when the energy required for biomass harvesting, electrode maintenance, pumping, and process control exceeds the additional energy recovered. The reviewed studies also show that integrated systems are more sensitive to operational complexity than single technologies. The superiority of integration depends on system design, process compatibility, scale, and the economic value of recovered products. The transition from single-technology optimization toward integrated wastewater-to-biofuels systems is supported by the temporal distribution of the reviewed literature. As shown in Fig. 3, the cited studies reveal a gradual expansion of research attention from anaerobic digestion and bioelectrochemical systems toward algal biorefineries, circular economy principles, techno-economic analysis, and life-cycle assessment.

**Fig. 4:**
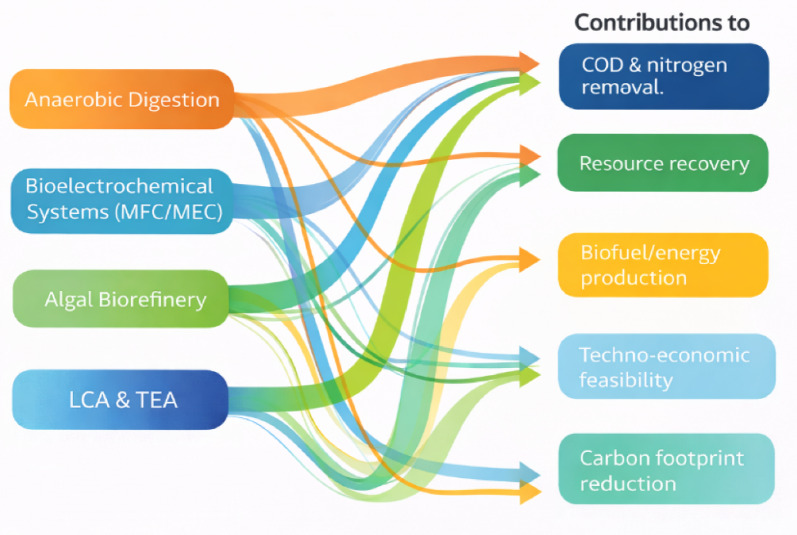
Diagram of the contribution of wastewater-to-biofuels technology to the achievement of SDG 6. **Source:** Primary Data (2026)

Figure 3 indicates that wastewater-to-biofuels research has expanded substantially after 2017, with increasing attention to algal biorefineries, circular economy approaches, techno-economic analysis, and life-cycle assessment. The figure is used to show the temporal and geographical distribution of the evidence base, rather than to repeat the conceptual argument on SDG 6. The relationship between integrated wastewater-to-biofuels technologies and SDG 6 is summarized in Fig. 4. The figure shows that anaerobic digestion, bioelectrochemical systems, and algal biorefineries contribute to sustainability not only through energy production, but also through pollutant reduction, nutrient recovery, economic feasibility, and carbon footprint mitigation.

Figure 4 functions as a synthesis diagram that links wastewater-to-biofuels technologies with SDG 6-related sustainability pathways. It shows that anaerobic digestion, bioelectrochemical systems, and algal biorefineries contribute through pollutant reduction, resource recovery, renewable energy generation, economic feasibility, and carbon footprint mitigation. To strengthen the analytical synthesis, the reviewed technologies were compared across single and integrated system configurations. As shown in Table 1, the comparison highlights where integration provides clear advantages, where single technologies remain more suitable, and under which operational conditions integration may become counterproductive.

**Table 1:** Comparative synthesis of single and integrated wastewater-to-biofuels systems

System configuration	Main strength		Main limitation	When the system performs better	When the system becomes counterproductive	TEA–LCA implication
Anaerobic digestion	Stable biogas production and high COD removal		Limited nutrient recovery and methane leakage risk	High-strength wastewater with stable organic loading	Low-strength wastewater without co-digestion or pretreatment	Economically mature, but climate benefits depend on methane control
Bioelectrochemical systems	Direct electricity or hydrogen recovery		Low power density, electrode cost, scaling difficulty	Wastewater with biodegradable organic substrates and stable microbial activity	High internal resistance, unstable biofilm, costly electrode replacement	Environmental benefits may decline if material and energy inputs are high
Algal biorefinery	Nutrient recovery and biomass-based bioproducts		High harvesting and dewatering costs	Nutrient-rich wastewater with adequate light and CO₂ availability	Low biomass productivity or high downstream energy demand	LCA benefits depend on low-energy harvesting and high-value biomass use
AD–BES integration	Additional energy recovery from residual organics		Reactor complexity and higher monitoring needs	Digestate or effluent still contains recoverable organic matter	Energy gain is lower than additional operational energy	Potentially improves energy balance, but CAPEX and electrode costs are critical
AD–algae integration	Biogas production combined with nutrient recovery		Land, light, and biomass processing constraints	Digestate contains sufficient nitrogen and phosphorus for algal growth	Harvesting energy exceeds biomass value or nutrient uptake is unstable	Strong circular economy potential if biomass is converted into high-value products
BES–algae integration	Coupling electrochemical recovery with nutrient assimilation		Limited full-scale evidence	Systems designed for low-carbon polishing and nutrient capture	Poor electrochemical performance or excessive operational control	Requires careful TEA–LCA validation before scale-up
AD–BES–algae integration	Multi-output recovery of energy, nutrients, and bioproducts		Highest technical and managerial complexity	Medium- to large-scale facilities with stable influent and product valorization pathways	Small-scale systems, unstable wastewater, high energy demand, or weak market for by-products	Highest sustainability potential, but only under optimized design and supportive policy conditions

Source: Primary Data (2026)

Table 1 shows that integrated wastewater-to-biofuels systems provide the strongest sustainability potential when process compatibility, substrate availability, nutrient balance, and product valorization are aligned. Anaerobic digestion remains advantageous for stable organic matter conversion and biogas production, while bioelectrochemical systems offer additional value through direct electricity or hydrogen recovery. Algal biorefineries strengthen nutrient recovery and diversify bioproduct outputs. The comparative synthesis also indicates that integration is not automatically superior to single technologies. Integrated systems may become less feasible when wastewater strength is low, influent characteristics are unstable, downstream energy demand is high, or the market value of recovered products is insufficient. The feasibility of AD–BES–algae integration depends on a balance between technical synergy, operational complexity, economic viability, and life-cycle environmental performance. Table 1 confirms that integration offers clear advantages when AD, BES, and algal biorefineries operate in a complementary manner. Its benefits are most evident in systems with sufficient organic load, recoverable nutrients, stable operation, and viable product valorization. Integration may become counterproductive when the additional energy demand, capital cost, process control, or downstream processing burden exceeds the value of recovered energy and bioproducts. This finding indicates that integrated systems must be evaluated through both technical performance and TEA–LCA feasibility. To contextualize this comparative synthesis, Table 2 presents the evolution of wastewater-to-biofuels research from single-technology approaches to integrated systems.

**Table 2:** Evolution of wastewater-to-biofuels research focus from single technologies to integrated systems

Period	Dominant focus of research	Study characteristics	Example theme
2005–2009	Anaerobic digestion (AD)	Optimization of biogas production and COD reduction	Methane yield, sludge digestion, energy recovery
2010–2013	Bioelectrochemical systems (BES)	MFC/MEC development and electricity/hydrogen production	Bioelectricity, microbial communities
2014–2016	Resource recovery and circular economy	Transformation of WWTP into a resource recovery facility	Nutrient recovery, phosphorus recovery
2017–2019	Algal biorefinery integration	Integration of microalgae with wastewater treatment	Algae cultivation, biodiesel production
2020–2022	Sustainability assessment	Integration of TEA and LCA in bioenergy systems	Carbon footprint, techno-economic feasibility
2023–2025	Integrated wastewater-to-biofuels systems	AD–BES–algae integrated system model based on SDG 6	Hybrid systems, circular bioeconomy

Source: Primary Data (2026)

In the early period, research on wastewater-to-biofuels was dominated by the optimization of anaerobic digestion as the main technology in energy recovery from wastewater. [1, 7, 67] show that the regulation of temperature and hydraulic retention time plays an important role in improving the stability of methane production. [2, 33] emphasize that energy conversion from wastewater organic substrates has significant potential as a renewable energy source. [22, 41, 60] identify inhibition factors such as ammonia accumulation that affect the efficiency of anaerobic digestion. [6, 19, 59] comprehensively describe the principles and potential of active sludge digestion in increasing biogas yield. [17, 29, 39, 70] emphasize that wastewater treatment plants have the potential to become an energy-positive system if the chemical energy content of waste is optimally utilized.

Entering the period 2010–2013, the focus of research began to shift to the development of bioelectrochemical systems (BES) such as microbial fuel cells (MFC) and microbial electrolysis cells (MEC). [23–25, 42, 49] review MFC’s opportunities to generate electricity directly from wastewater while reducing organic burdens. [16, 45, 64] show that MEC is able to produce hydrogen with competitive energy efficiency compared to conventional methods. [21, 65, 71] expand the water–energy nexus framework that integrates energy systems and wastewater management in a single sustainability perspective. [11, 44] emphasize the importance of life-cycle assessment in evaluating the environmental impact of waste treatment technology. [46, 58] also showed that the stability of the electrogenic microbial community plays an important role in improving the power density and performance of the BES system.

The 2014–2016 period was marked by a paradigm transformation toward a resource recovery and circular economy approach. [20, 66] expressly state that wastewater treatment plants must be transformed into resource recovery facilities. [75] demonstrate that modern installations have the potential to produce clean energy beyond their operational consumption. [30] introduce the importance of techno-economic analysis in assessing the feasibility of investment in mud-based biogas systems. [38, 63] emphasize the urgency of phosphorus recovery as an increasingly limited strategic resource. [12, 35] articulate the circular economy as a new paradigm of sustainability that promotes the integration of energy and nutrient recovery in wastewater treatment systems.

In the 2017–2021 period, the integration of microalgae and sustainability approaches was increasingly strengthened in the literature. [27, 61] show that microalgae are effective in removing nitrogen and phosphorus while also producing lipid biomass for biodiesel. [5, 26] emphasize the importance of economic evaluation in an integrated anaerobic–algae system. [9, 18] research on the use of anaerobic digestate as a microalgae culture medium to improve carbon efficiency. [72, 73] LCA on wastewater-based biofuel production lines highlights the impact of greenhouse gas emissions. [3, 10, 53] show that a bioelectrochemical–anaerobic hybrid system is able to improve clean energy efficiency and improve overall environmental performance.

The latest period 2022–2025 shows consolidation toward an integrated system based on SDG 6 and a circular bioeconomy. [37, 55] explicitly link the wastewater-to-bioenergy system to the achievement of SDG 6 targets through the water–energy–nutrient nexus approach. [40, 57] show that the multi-technology integration of AD–BES–algae improves the efficiency of simultaneous energy and nutrient recovery. [54] examine the technical integration and evaluation of LCAs in city-scale wastewater-to-biofuels systems. Rahman et al. [54] emphasized the importance of policy integration and the circular economy in the implementation of sustainable technology. [62] develop an integrated system model that combines energy efficiency, nutrient recovery, and carbon emission mitigation in one comprehensive framework that directly supports the achievement of SDG 6.

Based on Table 3, the distribution of technology focus in the literature shows that each wastewater-to-biofuels approach has different but complementary output characteristics, evaluation methods, and implementation challenges. Anaerobic digestion remains the most established technology with the main outputs in the form of biogas (CH₄) and digestate, which are generally analyzed through energy balance and techno-economic analysis (TEA) approaches, although it still faces the issue of conversion efficiency and long-term process stability. Bioelectrochemical systems such as MFC and MEC offer innovations in electricity and biohydrogen production, with evaluations based on power density analysis and life-cycle assessment (LCA), but are constrained by commercial scale and high cost of electrode materials. Meanwhile, algal biorefineries contribute to the production of biodiesel, biomass, and bioplastics, which are analyzed through TEA, LCA, as well as carbon assessment, but still face challenges in the cost of cultivation and biomass harvesting processes. Hybrid systems that integrate various technologies offer multi-product outputs of energy and nutrient recovery, with an integrated TEA–LCA evaluation approach, although the complexity of technical and managerial integration is a major challenge. This table confirms that the success of achieving SDG 6 is highly dependent on technological synergy that is not only technically efficient, but also economically feasible and environmentally sustainable.

**Table 3:** Distribution of technology focus and analytical approaches in the literature

Key technologies	Energy/bioproduct output	Analytical approach used	Key challenges
Anaerobic digestion	Biogas (CH₄), digestate	Energy balance, TEA	Conversion efficiency and process stability
Bioelectrochemical systems	Electricity, biohydrogen	Power density analysis, LCA	Commercial scale and electrode cost
Algal biorefinery	Biodiesel, biomass, bioplastics	TEA, LCA, carbon assessment	Cultivation and harvesting costs
Hybrid systems	Multi-output (energy + nutrients)	Integrated TEA–LCA	Complexity of system integration

Source: Primary Data (2026)

Based on Table 4, the contribution of the wastewater-to-biofuels system to the achievement of SDG 6 targets shows a comprehensive linkage between technical, environmental, and economic aspects in one integrative framework. In the dimension of improving water quality, the integrated system is able to significantly reduce the concentration of COD, nitrogen, and phosphorus, thereby contributing to the reduction of the risk of eutrophication and pollution of water bodies. In terms of resource efficiency, energy and nutrient recovery allow the transformation of wastewater treatment plants into energy-positive wastewater treatment plants that not only treat waste, but also produce energy and value-added materials. In the context of climate change mitigation, the production of renewable energy from biogas, biohydrogen, and biodiesel contributes to reducing greenhouse gas emissions. This approach also strengthens the principle of the circular economy through the conversion of waste into bioproducts that have economic value, while increasing water and energy security through the integration of the water–energy nexus.

**Table 4:** Contribution of wastewater-to-biofuels to SDGs target 6

SDGs dimension 6	Integrated system contribution	Impact indicators
Improved water quality	Reduction of COD, nitrogen, phosphorus	Decrease in eutrophication
Resource efficiency	Energy and nutrient recovery	Energy-positive WWTP
Climate change mitigation	Renewable energy production	Reduction of GHG emissions
Circular economy	Conversion of waste into bioproducts	Economic added value
Water and energy resilience	Water-energy nexus integration	Sustainable resource management

Source: Primary Data (2026)

**Fig. 5:**
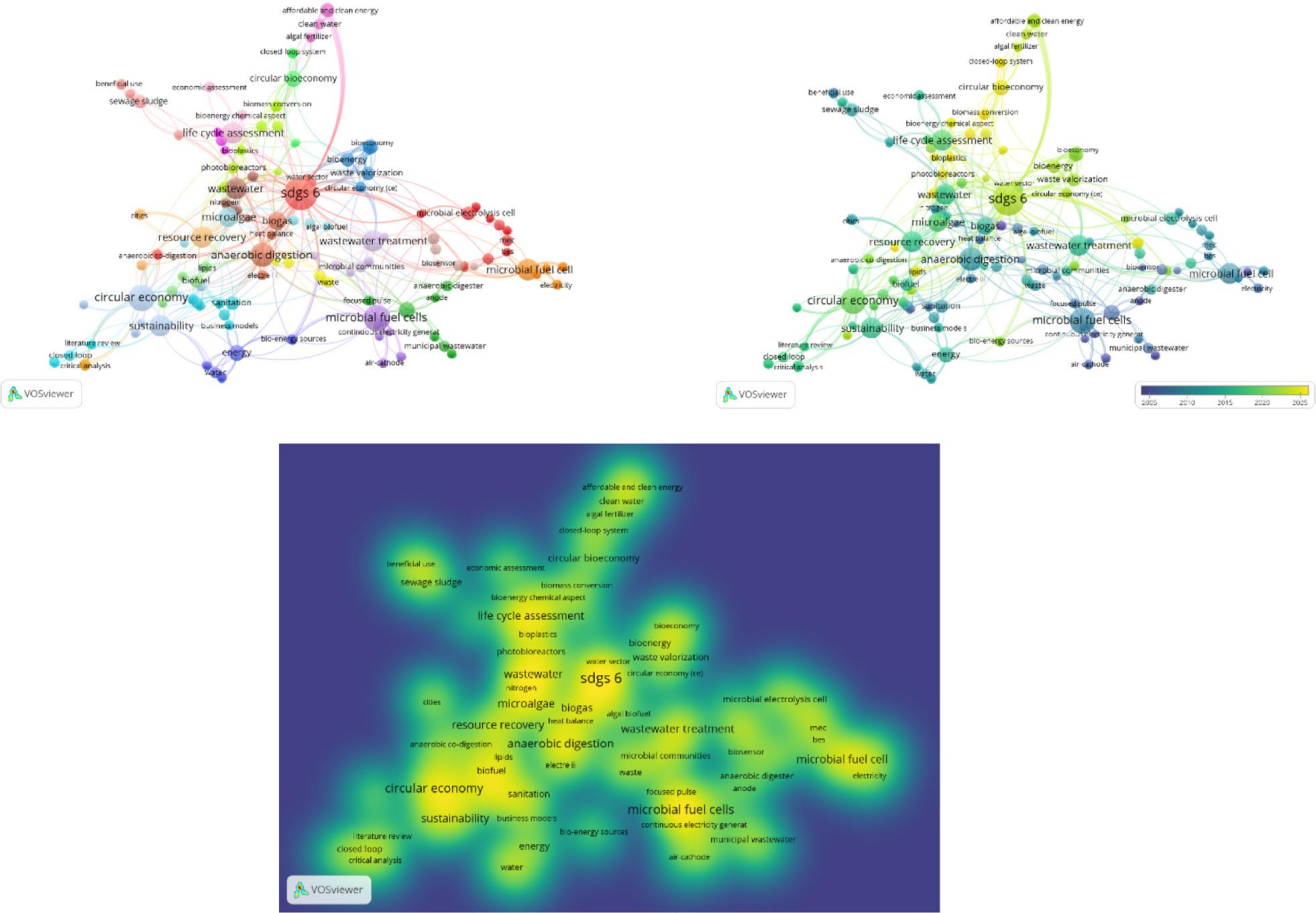
Bibliometric mapping of wastewater-to-biofuels keywords based on co-occurrence and temporal trends (2005–2025). **Source:** VOSviewer Output (2026)

Figure 5 presents the bibliometric structure of the reviewed literature. The co-occurrence network shows how keywords such as anaerobic digestion, microbial fuel cells, circular economy, life-cycle assessment, and resource recovery are connected within the field. This figure complements Figs. 3 and 4 by showing the intellectual structure of the literature, rather than the temporal distribution or SDG 6 contribution pathway. Based on Fig. 5, the bibliometric visualization shows the network structure of the keywords that make up several major clusters in the wastewater-to-biofuels research period 2005–2025. On the co-occurrence map, it can be seen that terms such as anaerobic digestion, microbial fuel cells, circular economy, life-cycle assessment, and SDG 6 occupy a central position that connects various research subthemes. This indicates that the research focus is no longer partial, but rather moving toward integration between energy recovery technologies, sustainability evaluation, and circular economy frameworks. The temporal overlay shows a shift in trend from the dominance of anaerobic digestion and microbial fuel cells in the early period to the integration of the concepts of circular bioeconomy, resource recovery, and life-cycle assessment in the more recent period. The density map confirms that the keywords with the highest intensity are centered on a combination of anaerobic digestion, microbial fuel cells, circular economy, and sustainability, which shows the concentration of the literature on technology synergy and sustainability.

This visualization shows the evolution of the research paradigm from a single-technology approach to an integrated system based on the water–energy–nutrient nexus that is explicitly linked to the achievement of SDG 6. The density and interconnectedness between keywords indicate that the current literature increasingly emphasizes the importance of evaluation based on techno-economic analysis and life-cycle assessment to ensure the feasibility and environmental impact of the developed system. Thus, this image not only represents a bibliometric pattern, but also illustrates a conceptual transformation in wastewater-to-biofuels research, from simply waste treatment to a circular bioeconomy model oriented toward global sustainability. To avoid conceptual redundancy, SDG 6, circular economy, and the water–energy–nutrient nexus are positioned differently across the manuscript. SDG 6 is used as the policy frame, circular economy as the resource recovery principle, and the nexus perspective as the analytical lens for linking water treatment, energy production, and nutrient recovery.

### Discussion

The synthesis indicates that the main contribution of integrated wastewater-to-biofuels systems lies in their ability to combine organic matter conversion, nutrient recovery, renewable energy production, and environmental impact reduction within a single resource recovery framework. The discussion therefore focuses on the comparative performance, TEA–LCA trade-offs, and implementation conditions of single and integrated technologies, rather than restating the general SDG 6 and circular economy rationale.

#### Technical performance evaluation of wastewater-to-biofuels technology

The evaluation of the technical performance of wastewater-to-biofuels technology is the main foundation for determining the feasibility of transforming conventional wastewater treatment plants into sustainable energy and resource recovery systems. In general, the three main technological approaches that dominate the literature are anaerobic digestion (AD), bioelectrochemical systems (BES) such as microbial fuel cells (MFC) and microbial electrolysis cells (MEC), and algal biorefinery [47]. All three have different operational characteristics, energy conversion mechanisms, and performance indicators, but conceptually have the same goal, which is to maximize the recovery of chemical energy contained in wastewater while significantly reducing the pollutant load [6]. Technical evaluation in this context is not only limited to energy production, but also included the efficiency of chemical oxygen demand (COD) reduction, nutrient recovery, process stability, sensitivity to fluctuations in organic loads, as well as system integration on a broader operational scale.

In anaerobic digestion technology, technical performance is generally measured through methane production parameters (CH₄ yield), COD reduction rate, pH stability, and hydraulic retention time (HRT). The literature shows that AD is capable of reducing COD by up to 70–90% depending on the characteristics of the substrate and the reactor configuration, with methane production ranging from 0.2 to 0.35 m^3^ CH₄ per kg of degraded COD. This efficiency makes AD the most mature technology and widely implemented commercially in wastewater sludge treatment [36]. The technical performance of AD is greatly influenced by inhibiting factors such as ammonia accumulation, temperature fluctuations, and an imbalance between acidogenic and methanogenic bacteria. In addition, at low organic loads such as diluted domestic wastewater, methane production is often not high enough to achieve energy-positive wastewater treatment plant conditions without a pretreatment or co-digestion strategy [11]. Thus, although AD has the advantages of technological stability and maturity, its technical challenge lies in optimizing energy conversion under dynamic operational conditions.

Bioelectrochemical systems (BES) utilize electrogenic microorganisms to transfer electrons directly to the electrode, thus producing electricity (MFC) or hydrogen (MEC). The technical performance of BES is usually measured through power density, coulombic efficiency, and degradation rate of organic matter. Some studies have shown that MFCs can achieve power densities between 1–2 W/m^2^ under laboratory conditions, with a COD reduction of up to 80% [30]. Meanwhile, MECs are capable of producing hydrogen with relatively high energy efficiency when combined with low external voltages. Although BES theoretically offers the advantage of direct energy production without the combustion stage, the technical challenges lie in the internal resistance of the system, the cost of electrode materials, biological fouling, as well as the limitations of scale [61]. The stability of the electrogenic microbial community is also a crucial factor in maintaining long-term performance. Therefore, from a technical perspective, BES has high potential but still requires the development of materials and reactor design to achieve a level of commercialization equivalent to AD.

Algal biorefinery offers a different approach by utilizing nutrients (nitrogen and phosphorus) in wastewater as a medium for microalgae growth. The technical performance of this technology is assessed through biomass productivity (g/L/day), lipid content (% dry weight), and nutrient absorption efficiency [5]. The literature shows that microalgae are able to remove up to 70–95% of nitrogen and phosphorus from wastewater, while producing biomass with a lipid content of up to 20–40% depending on the species and culture conditions [26]. This biomass can then be processed into biodiesel, bioplastics, or high-value bioactive products. The main technical constraints lie in the energy requirements for the harvesting and dewatering processes, sensitivity to other microbial contamination, as well as light and temperature fluctuations in open systems. Thus, while the potential for nutrient recovery and biofuel production is promising, the clean energy efficiency of algae systems is highly dependent on downstream process optimization.

Technical evaluation shows that the combination of AD–BES–algae can produce significant synergies. Digestate from AD can be used as a source of nutrients for algae culture, while BES can utilize organic residues to promote additional energy recovery. This hybrid system has the potential to improve total energy recovery efficiency, improve process stability, and reduce the overall carbon footprint. Some experimental models show that the integration of AD with microalgae can increase carbon recovery by more than 50% compared to a single system. The complexity of operational controls, the need to synchronize process parameters, and the increased investment costs are major challenges in the technical implementation of integrated systems. Comparatively AD excels in technological stability and maturity, BES excels in direct energy conversion innovation, while algae excel in nutrient recovery and bioproduct diversification. Technical evaluations show that no single technology is completely optimal independently in meeting all dimensions of sustainability. The integrated systems approach is becoming increasingly relevant within the framework of the water–energy–nutrient nexus. The transformation of wastewater treatment plants into resource recovery systems requires a combination of technologies that are able to optimize energy efficiency, process stability, and nutrient recovery simultaneously.

The evaluation of the technical performance of wastewater-to-biofuels technologies shows that significant progress has been made in the past two decades, but challenges remain in terms of scalability, clean energy efficiency, and system integration. The technological ability to support the achievement of SDG 6 depends not only on the efficiency of pollutant reduction, but also on the capacity to generate renewable energy and recover resources economically. Thus, the development of an integrated system model that combines the advantages of each technology is a strategic direction in realizing future wastewater treatment plants that are sustainable and based on a circular economy.

#### Research trends and systems integration models in the literature

The development of the wastewater-to-biofuels literature in the last two decades not only shows an increase in the number of publications, but also reflects an epistemological transformation in the way scientists view wastewater itself. If in the early phases wastewater is positioned as an environmental burden that must be minimized, then the contemporary literature places it as a strategic resource node in a circular bioeconomic system [72]. This shift indicates a shift in orientation from a process unit-based reductionistic approach to a nexus-based systemic approach. This means that research is no longer pursuing local efficiency in a single technology, but rather seeking to understand how the flow of carbon, energy, and nutrients can be optimized simultaneously in an integrated configuration. This transformation reflects the integration of environmental engineering, bioenergy, circular economy, and sustainability policies in one more complex analytical framework.

The dynamics of research trends also show a shift from exploring individual technology performance to exploring system architecture. In the recent literature, research questions no longer focus on "how much energy can be generated", but on "how the design of the system can maximize the total value generated from waste." This marks a shift from single-output optimization to multi-output optimization. System configurations are starting to be designed to integrate energy, nutrient and biomass recovery simultaneously, creating systems with higher functional redundancy and resilience [53]. This perspective shows that complexity is no longer seen as an obstacle, but rather as an opportunity to improve the total efficiency of the system through synergies between processes.

The trend of systems integration in the literature reflects the increasing adoption of systems thinking and cross-disciplinary approaches. The integration between biological, electrochemical, and phototrophic technologies is no longer seen as a mechanical combination, but rather as a mutually reinforcing metabolic and material interaction. Digestate is not only considered as a residue, but as a nutrient medium; electrons are not only considered as a by-product of metabolism, but as an energy commodity; and biomass is not only considered as secondary waste, but as raw materials for green industries [10]. Thus, the model of integration that is developing in the literature shows an ontological shift: from a processing system to a production system. This shift implies changes in infrastructure design, investment patterns, and resource management policies.

An in-depth interpretation of research trends also shows that system integration is increasingly influenced by external pressures in the form of global targets such as the SDGs and decarbonization commitments. This prompted the literature to discuss not only technical efficiency, but also the compatibility of systems with long-term sustainability frameworks. In other words, technology integration is part of the energy transition narrative and circular economy transformation. Recent literature shows that the success of an integrated system is determined not only by the parameters of the reactor, but also by its suitability with social, economic, and policy contexts. The emerging model of integration is no longer universal, but rather contextual and adaptive to regional characteristics.

Conceptually this trend suggests that wastewater-to-biofuels research is moving toward a stage of systemic consolidation, where various technological approaches are no longer treated as competing alternatives, but rather as complementary components in a single sustainable production ecosystem. Contemporary literature confirms that the future of wastewater treatment plants is not only efficient in removing pollutants, but also capable of serving as nodes for energy production, nutrient recovery, and carbon emission reduction simultaneously. Thus, research trends and systems integration models in the literature reflect not only technical advances, but also paradigm transformations toward the reconstruction of water infrastructure as an integral part of the global circular bioeconomy.

#### Integrated TEA–LCA synthesis and decision framework

Techno-economic analysis and life-cycle assessment provide complementary evaluation lenses for determining the feasibility of wastewater-to-biofuels and bioproduct systems. TEA assesses whether a technology configuration can generate economic value through acceptable investment, operating cost, revenue, and payback performance, while LCA evaluates whether the same configuration reduces environmental burdens across the system life cycle, including greenhouse gas emissions, eutrophication potential, cumulative energy demand, and resource depletion [26, 30]. TEA and LCA should not be treated as separate post-evaluation tools, but as an integrated decision framework for comparing single and hybrid wastewater-to-biofuels systems [5, 23, 52].

In single-technology systems, TEA–LCA outcomes are often easier to interpret because the system boundary, input–output flows, and operational units are relatively limited. Anaerobic digestion generally provides more predictable techno-economic performance due to technological maturity and established biogas utilization pathways [30]. Bioelectrochemical systems offer additional energy recovery potential, yet their economic feasibility is strongly affected by electrode costs, reactor materials, and power density limitations [12, 34, 42]. Algal biorefineries provide environmental benefits through nutrient uptake and biomass valorization, yet their economic and environmental performance may decline when harvesting, dewatering, and downstream processing require high energy input [4, 9, 18].

In integrated systems, TEA–LCA interpretation becomes more complex because additional process units can simultaneously improve and reduce system feasibility. Integration may improve net present value and reduce environmental impacts when residual streams from one technology become useful inputs for another process, such as digestate utilization for algal cultivation or residual organics for bioelectrochemical conversion [10, 26, 40]. Integration may increase capital expenditure, operational expenditure, process-control requirements, and indirect emissions. The decision to integrate AD, BES, and algal biorefineries should be based on a comparative assessment of economic and environmental trade-offs, rather than on the assumption that integration is always superior [54, 62]. To clarify how TEA and LCA indicators are jointly used in evaluating single and integrated wastewater-to-biofuels systems, Table 5 maps the main economic and environmental indicators and explains how each indicator informs technology selection and system integration decisions.

**Table 5:** Mapping of TEA and LCA indicators for wastewater-to-biofuels system evaluation

Evaluation dimension	Key indicator	Main function in system evaluation	Relevance to single technologies	Relevance to integrated systems
TEA	Capital expenditure (CAPEX)	Measures initial investment for reactors, electrodes, algal ponds, harvesting units, and auxiliary infrastructure	Lower and more predictable for mature systems such as anaerobic digestion	Usually higher due to multiple process units and integration infrastructure
TEA	Operational expenditure (OPEX)	Measures recurring costs for energy, labor, chemicals, maintenance, biomass harvesting, and reactor control	Easier to estimate because operational boundaries are narrower	May increase due to pumping, monitoring, process control, electrode maintenance, or dewatering
TEA	Net present value (NPV)	Assesses long-term financial viability by comparing investment cost and future revenue	Depends mainly on one product stream, such as biogas or biomass	Can improve when multiple products generate revenue, such as biogas, hydrogen, biomass, and nutrients
TEA	Internal rate of return (IRR)	Indicates investment attractiveness compared with alternative uses of capital	More stable for mature and commercially available technologies	Sensitive to market value of recovered products and scale of implementation
TEA	Payback period	Measures how quickly investment costs can be recovered	Shorter when technology is mature and energy output is stable	May be longer because integrated systems usually require higher upfront investment
TEA	Product valorization	Evaluates the economic value of recovered energy, nutrients, and bioproducts	Limited to one dominant output	Stronger when multiple outputs can be monetized effectively
LCA	Global warming potential (GWP)	Measures greenhouse gas emissions across the life cycle	AD can reduce fossil energy use but may be affected by methane leakage	Integration can reduce net GWP if recovered energy offsets external energy demand
LCA	Eutrophication potential	Evaluates nutrient-related environmental impacts	Limited nutrient recovery in AD and BES alone	Lower when algal systems recover nitrogen and phosphorus effectively
LCA	Cumulative energy demand	Measures total direct and indirect energy use	Lower in simpler systems with fewer process units	May increase if integration requires intensive pumping, harvesting, or control systems
LCA	Carbon footprint reduction	Evaluates avoided emissions from renewable energy and resource recovery	Depends on the substitution of fossil energy or synthetic products	Stronger when multiple outputs replace fossil-based energy, fertilizers, or materials
LCA	Resource depletion	Assesses pressure on non-renewable materials and resources	Lower in low-material systems	May increase if electrodes, membranes, or specialized materials are required
LCA	Life-cycle trade-off	Identifies environmental burden shifting across process stages	Easier to detect due to simpler boundaries	More critical because benefits in one unit may create burdens in another

Table 5 shows that TEA and LCA provide different but complementary forms of evidence. TEA identifies whether a system is economically feasible through investment, operating cost, revenue, and payback indicators, while LCA determines whether the same system reduces environmental burdens across the life cycle. In single technologies, feasibility is often easier to assess because costs, outputs, and environmental impacts are concentrated in one dominant process. In integrated systems, feasibility depends on whether additional outputs and avoided environmental burdens are sufficient to compensate for higher CAPEX, OPEX, process complexity, and indirect emissions. This synthesis indicates that AD–BES–algae integration is most favorable when the system can simultaneously recover energy, nutrients, and value-added biomass with limited additional energy input. Integration becomes less attractive when additional units increase life-cycle burdens without generating proportional economic or environmental benefits.

**Fig. 6:**
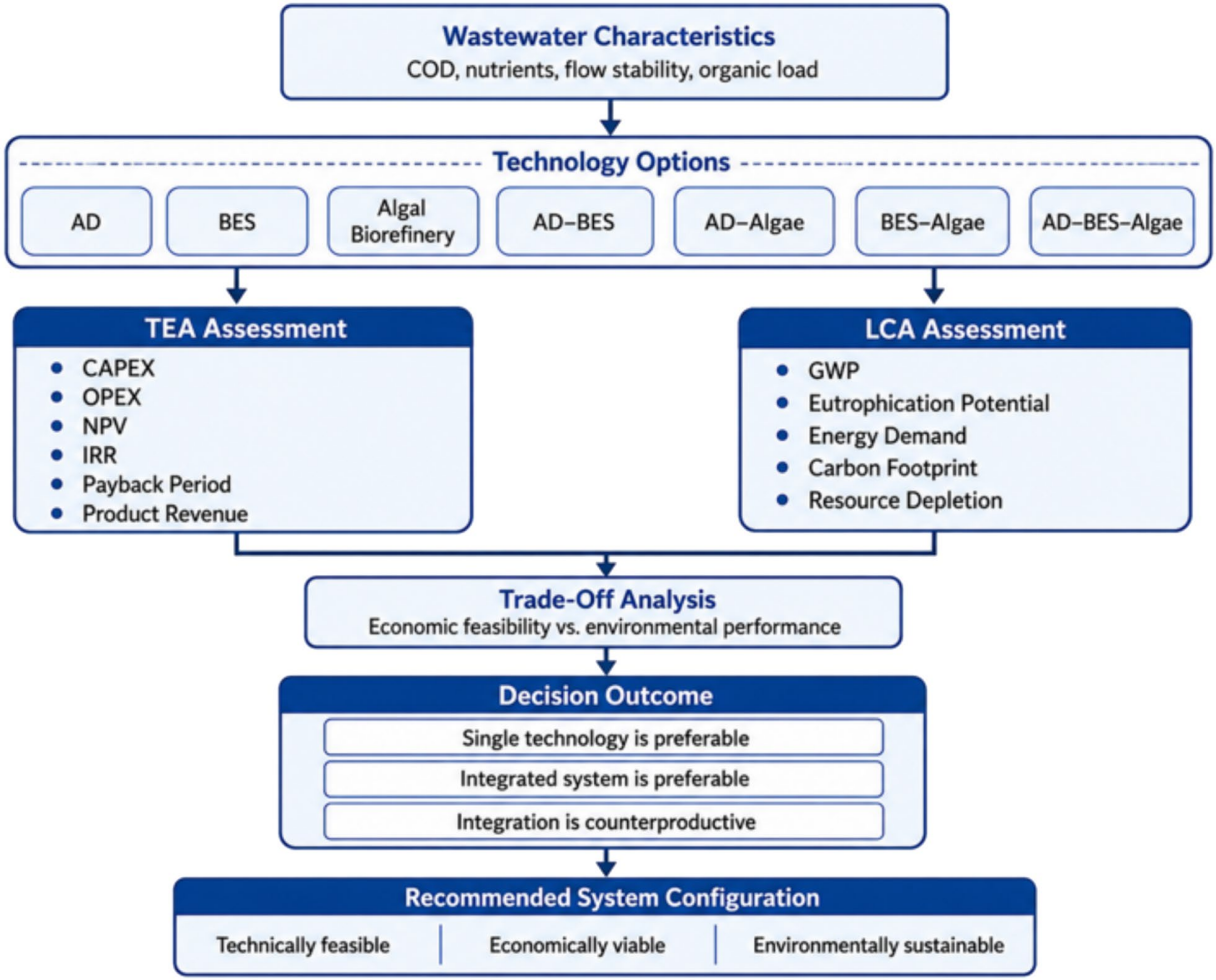
Conceptual TEA–LCA decision framework for selecting single or integrated wastewater-to-biofuels systems

Based on the indicator mapping in Table 5, a conceptual TEA–LCA decision framework was developed to show how economic and environmental evidence can be jointly used to select the most appropriate wastewater-to-biofuels configuration. The framework is presented in Fig. 6.

Figure 6 illustrates that technology selection should begin with wastewater characteristics, including organic load, nutrient concentration, flow stability, and substrate biodegradability. These characteristics determine whether a single technology or an integrated configuration is technically appropriate. TEA is then used to assess investment feasibility, operating costs, revenue potential, and payback performance, while LCA evaluates greenhouse gas emissions, eutrophication potential, energy demand, carbon footprint, and resource depletion. The final decision depends on the trade-off between economic feasibility and environmental performance. A single technology is preferable when it provides stable performance with lower cost and lower system complexity. An integrated system is preferable when additional recovery pathways generate sufficient economic and environmental benefits. Integration becomes counterproductive when additional process units increase CAPEX, OPEX, energy demand, or life-cycle emissions without proportional gains in resource recovery or product value.

#### Critical synthesis of recent data-driven evidence

Recent research outputs indicate that the performance of wastewater-to-biofuels systems cannot be assessed only through technological potential or conceptual alignment with the circular economy. The reviewed studies show that actual feasibility is strongly shaped by data-driven parameters, including organic loading rate, nutrient concentration, biomass productivity, electrode performance, energy input, product yield, carbon intensity, and market value of recovered products [26, 30]. This means that academic discussion on wastewater-to-biofuels systems should move beyond whether AD, BES, and algal biorefineries are technically promising, toward a more critical examination of when these technologies produce measurable economic and environmental benefits.

Evidence from recent TEA–LCA studies shows that the same technology may produce different sustainability outcomes depending on system boundaries, process assumptions, allocation methods, energy sources, and product substitution scenarios [23]. Wastewater-derived bioresource production may offer environmental benefits when recovered products substitute fossil-based energy, synthetic fertilizers, or external materials, yet these benefits can decline when the process requires high electricity input, intensive chemical use, or complex downstream processing [8]. Recent studies on microalgal and wastewater-based biorefineries also show that product diversification can improve economic feasibility, but only when biomass productivity, harvesting efficiency, and marketable co-products are sufficiently strong [8, 28].

The evidence also suggests that integrated systems are not automatically more advanced than single technologies. AD-based systems often show stronger readiness because of technological maturity and established biogas utilization pathways [30]. BES-based systems remain scientifically promising but are constrained by electrode cost, low power density, scale-up uncertainty, and long-term operational stability [12, 34, 42]. Algal biorefineries provide strong nutrient recovery potential, yet their feasibility is frequently limited by harvesting, dewatering, contamination risk, and variable biomass conversion efficiency [4, 9, 18, 28]. The depth of the current evidence supports a conditional rather than universal argument for integration.

A critical reading of the recent literature indicates that integration becomes academically and practically meaningful when one process improves the input condition, energy balance, or environmental performance of another process. For instance, AD can stabilize organic waste and generate digestate that may support algal cultivation, while BES can recover additional energy from residual biodegradable substrates [10, 26, 40]. This synergy is weakened when the added process units increase CAPEX, OPEX, energy demand, maintenance burden, or life-cycle emissions without proportional gains in recoverable products [54, 62]. Thus, recent data-driven evidence supports a trade-off-based interpretation: integration should be selected only when it improves net sustainability performance across technical, economic, and environmental dimensions.

This critical synthesis strengthens the review’s contribution by showing that the future development of wastewater-to-biofuels systems depends less on the availability of individual technologies and more on evidence-based configuration. The most promising research direction is not simply to combine AD, BES, and algal biorefineries, but to identify specific wastewater characteristics, operational scales, and product recovery pathways under which integration generates measurable value [28, 62]. This perspective advances the discussion from broad technological mapping to a more rigorous assessment of recent empirical and modeling evidence. To make the evidence synthesis more explicit, Table 4 summarizes recent data-driven findings and their critical implications for selecting single or integrated wastewater-to-biofuels systems.

Table 6 shows that the strongest evidence does not support a simple hierarchy in which integrated systems are always superior to single technologies. Instead, the recent literature points to a context-dependent configuration logic. AD is more reliable when organic loading and biogas utilization are stable. BES is more suitable as an energy-recovery enhancer when residual biodegradable substrates are available. Algal biorefineries are most valuable when nutrient recovery and biomass valorization can offset harvesting and processing costs. Full integration offers the broadest circular economy potential, yet it requires stronger evidence of net benefits across TEA and LCA indicators. This finding responds to the need for a deeper academic discussion by interpreting recent research outputs through performance conditions, trade-offs, and implementation constraints.

**Table 6:** Critical synthesis of recent data-driven evidence in wastewater-to-biofuels systems

Evidence stream	Recent research output	Main data-driven finding	Critical interpretation	Implication for system selection
Anaerobic digestion	Studies on biogas production, COD reduction, and sludge valorization	AD shows relatively stable organic matter conversion and biogas recovery	AD is the most mature option, but methane leakage, low-strength wastewater, and limited nutrient recovery reduce its sustainability advantage	Preferable for high-strength organic wastewater and facilities with existing biogas infrastructure
Bioelectrochemical systems	Studies on MFC/MEC performance, electricity generation, and hydrogen recovery	BES can convert residual organic substrates into electricity or hydrogen	Scientific potential remains high, but low power density, electrode cost, and scale-up uncertainty limit practical readiness	Suitable as a complementary unit rather than a stand-alone commercial pathway
Algal biorefineries	Studies on nutrient recovery, biomass productivity, and bioproduct generation	Algae can recover nitrogen and phosphorus while producing biomass for biofuels and bioproducts	Feasibility depends on harvesting efficiency, dewatering energy, contamination control, and product valorization	Preferable for nutrient-rich wastewater and systems targeting high-value biomass products
AD–algae integration	Studies using digestate or wastewater nutrients for algal growth	Digestate can support algal cultivation and improve nutrient recovery	Integration is beneficial when nutrient loading and light availability support stable biomass productivity	Strong option when nutrient recovery and biomass valorization are prioritized
AD–BES integration	Studies combining anaerobic conversion and electrochemical recovery	BES can recover additional energy from residual biodegradable organics	Benefits may be reduced by electrode cost, reactor complexity, and maintenance requirements	Feasible when residual organics remain sufficient after AD and electrochemical recovery improves the net energy balance
Full AD–BES–algae integration	Modeling and review-based outputs on multi-stage recovery systems	Integrated systems can generate multiple outputs, including biogas, hydrogen/electricity, nutrients, and biomass	Sustainability benefits are conditional and may be offset by higher CAPEX, OPEX, energy demand, and operational complexity	Recommended only when TEA–LCA trade-offs show net economic and environmental gains
TEA–LCA evidence	Recent studies integrating economic and environmental modeling	Results are sensitive to system boundary, allocation method, energy source, and product substitution assumptions	No configuration is universally superior; feasibility depends on local infrastructure, energy mix, market value, and policy incentives	Technology choice should be guided by context-specific TEA–LCA decision analysis

#### Research gaps and development of integrative models based on the circular economy

The identification of research gaps in the wastewater-to-biofuels literature shows that although technological developments and sustainability evaluations have made significant progress over the past two decades, there are still substantial conceptual, methodological, and implementation gaps. Most studies focus on optimizing the performance of individual technologies under laboratory or pilot-scale conditions, while system integration at real operational scale is still limited:

1. The fragmentation gap of research approaches, in which anaerobic digestion, bioelectrochemical systems, and algal biorefineries are often studied separately without an integrative framework that brings together the flow of energy, carbon, and nutrients in a single systemic design. As a result, the potential for synergies between processes has not been fully utilized, and the system model developed tends to be partial and does not reflect the operational complexity of city-scale wastewater treatment plants.
2. The literature shows that most studies evaluate the performance of the technology in a relatively short observation period, so they have not been able to capture the dynamics of process stability, material degradation, and fluctuations in organic load in the long term. This is especially relevant for integrated systems that have higher operational complexity than a single technology. Without adequate longitudinal data, techno-economic evaluations and life-cycle assessments risk producing estimates that are overly optimistic or do not reflect real conditions. As such, there is an urgent need for full-scale demonstration-based research capable of testing the resilience of systems in real operational contexts.
3. Although techno-economic analysis (TEA) and life-cycle assessment (LCA) approaches are increasingly used, the integration of the two is often carried out separately without a systematic multi-criteria framework. Many studies emphasize economic feasibility without internalizing external environmental costs, or instead highlight environmental impacts without considering the financial realities of implementation. The absence of an integrated evaluation approach that considers social, policy, and governance aspects is also an important gap in the literature. In the context of a circular economy, sustainability is determined not only by technical and environmental efficiency, but also by social acceptance, regulations, as well as business models that support the adoption of technology.
4. Most of the literature comes from developed countries with relatively strong infrastructure and policy support, while studies in developing countries are still limited. In fact, the challenges of wastewater management and the need for renewable energy are often more urgent in areas with limited infrastructure capacity. Integrative models based on circular economies developed in the literature have not fully taken into account variations in local contexts, such as resource availability, energy tariff structures, or institutional capacity. This shows the need for an adaptive and contextual system design approach, rather than a universal, uniformly applied model.

Based on the identification of these gaps, the development of an integrative model based on the circular economy is a strategic agenda for further research. This model should be designed with the principle of a closed-loop system, where the flow of carbon, energy and nutrients is maximized in a single interconnected production cycle. Within this framework, anaerobic digestion functions as a primary energy conversion center, bioelectrochemical systems as an additional energy recovery booster, and algal biorefinery as a nutrient recovery mechanism as well as a producer of value-added bioproducts. This integration not only improves technical efficiency, but also creates output diversification that strengthens the overall economic viability of the system.

The proposed integrative model also needs to adopt a systems thinking approach, in which inter-component interactions are analyzed holistically. This includes mass and energy flow modeling, multi-objective optimization, and integration of TEA–LCA evaluation from the initial design stage. In addition, digitalization-based approaches and dynamic modeling can be used to predict system performance in various operational scenarios. Thus, integrative models are not only conceptual, but also operational and can be tested through simulations and real implementations. The development of this model must also take into account the creation of economic value from residues and by-products. The digestate can be utilized as organic fertilizer; algae biomass can be converted into bioplastics or green chemicals, while the electricity or hydrogen generated can be used to support the facility’s internal operations. This diversification allows for increased revenue streams while reducing dependence on one key product. This approach strengthens the economic resilience of the system to fluctuations in energy prices and market policies.

An integrative model based on a circular economy must also be linked to sustainable development targets, especially SDG 6. This means that the design of the system not only pursues energy efficiency, but also ensures improved water quality, eutrophication reduction, and sustainable sanitation access. The integration of the water–energy–nutrient nexus is a conceptual foundation that allows simultaneous and efficient management of resources. Thus, the model developed is not only a technical solution, but also a policy instrument to support the transition to sustainable water infrastructure. The synthesis of research gaps shows that the future of wastewater-to-biofuels research lies in adaptive systems integration, integrated multi-dimensional evaluation, and contextualization of implementation in various regions. The development of an integrative model based on the circular economy is a response to the fragmentation of the literature and the urgent need for systemic solutions. This model has the potential to reconstruct the role of wastewater treatment plants from just pollution-control facilities to strategic nodes in the global circular bioeconomy that support water, energy, and environmental security simultaneously.

The comparative synthesis indicates that integrated wastewater-to-biofuels systems should not be treated as universally superior to single technologies. Their sustainability advantage emerges only when process compatibility, resource availability, energy balance, and product valorization are aligned. Anaerobic digestion remains preferable for stable organic waste conversion; algal systems are more suitable for nutrient-rich streams, while bioelectrochemical systems offer added value when residual biodegradable substrates can be converted efficiently. A fully integrated AD–BES–algae configuration provides the broadest circular economy potential, but it also carries the highest risk of operational complexity, capital intensity, and unfavorable life-cycle trade-offs. The analytical contribution of this review lies in identifying the conditions under which integration creates synergy and the conditions under which it may reduce overall system feasibility.

## Conclusion

This systematic review shows that wastewater-to-biofuels and bioproducts systems have strong potential to support sustainable wastewater management through resource recovery, renewable energy generation, and environmental impact reduction. Anaerobic digestion remains the most mature pathway for biogas production and organic load reduction; bioelectrochemical systems offer additional electricity and hydrogen recovery, while algal biorefineries strengthen nutrient recovery and bioproduct diversification. The synthesis also shows that integrated systems are not universally superior to single technologies. Their feasibility depends on wastewater characteristics, process compatibility, operational scale, energy balance, product valorization, and TEA–LCA trade-offs. An integrated AD–BES–algae configuration is most promising when economic viability and environmental benefits are jointly achieved. Future studies should prioritize full-scale validation, dynamic TEA–LCA modeling, and context-specific implementation strategies, particularly in regions where wastewater treatment infrastructure and renewable energy access remain limited.

## Data Availability

No datasets were generated or analyzed during the current study.
